# The Degradation of Abdominal Aortic Prosthesis After 37 Years

**DOI:** 10.7759/cureus.77408

**Published:** 2025-01-14

**Authors:** Tomohiro Nakajima, Tsuyoshi Shibata, Yutaka Iba, Keishi Ogura, Nobuyoshi Kawaharada

**Affiliations:** 1 Department of Cardiovascular Surgery, Sapporo Medical University, Sapporo, JPN; 2 Division of Radiology and Nuclear Medicine, Sapporo Medical University, Sapporo, JPN

**Keywords:** abdominal aortic prosthesis, bleeding, damage, dilation, evar

## Abstract

An 85-year-old man underwent abdominal aortic aneurysm (AAA) repair with a synthetic graft at age 48. Sac enlargement, likely due to an endoleak, was observed during follow-up, and covered stents were placed in the graft limbs at age 80. Despite this, the sac continued to enlarge, leading to a referral to our institution. Imaging revealed an endoleak from an uncovered graft section. An EXCLUDER® stent graft (W. L. Gore & Associates, Inc., Flagstaff, AZ) was placed to cover the graft entirely, resolving the endoleak. The patient was discharged on postoperative day 5. This case highlights the rare failure of a synthetic graft after 37 years.

## Introduction

Synthetic grafts, which feature long-term durability and reduced perioperative risks, have significantly improved the surgical treatment of abdominal aortic aneurysms (AAA) [1,2]. However, late complications including graft failure, although rare, can occur decades after implantation [3]. These complications, such as structural degradation, endoleaks, or infections, may lead to aneurysm sac expansion or rupture, posing serious risks to patients. Reports of graft failure occurring more than 35 years after surgery are exceedingly uncommon, making such cases critical for advancing our understanding and improving the management and long-term outcomes of vascular surgery [4].

## Case presentation

**Figure 1:**
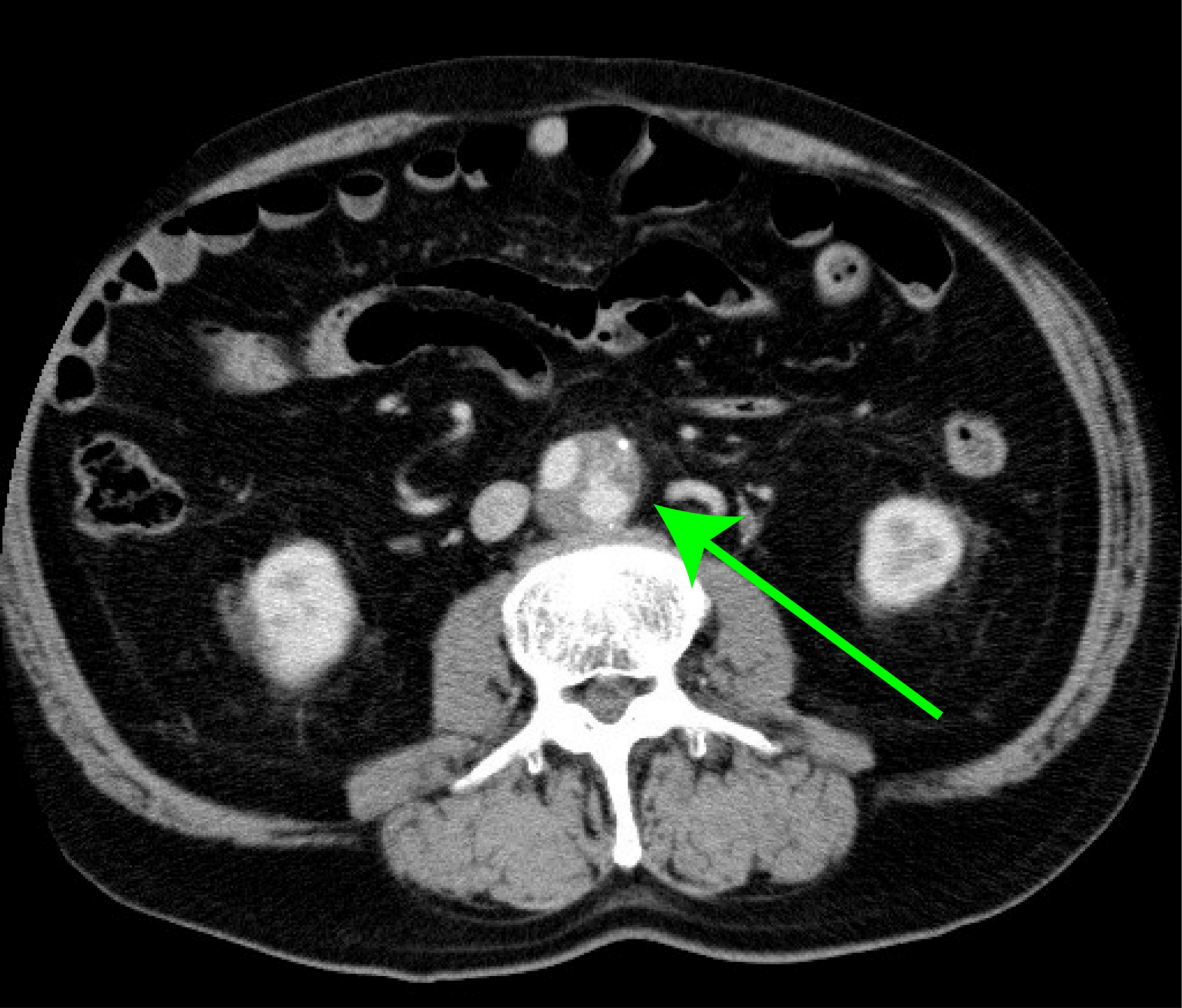
Preoperative computed tomography image. Contrast-enhanced computed tomography image taken at 25 years post-implantation revealing the synthetic graft within the aortic sac containing contrast leakage (green arrow).

At 48 years of age, the patient underwent synthetic graft replacement for an AAA via a midline laparotomy at another hospital. The details of the graft type were unavailable. A contrast computed tomography scan taken 25 years later showed a leak from the artificial blood vessel (Figure 1).

**Figure 2:**
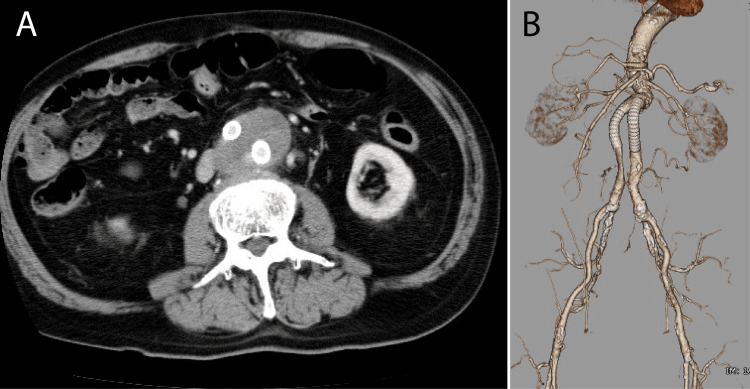
Contrast computed tomography image taken 32 years after artificial blood vessel replacement surgery in which a covered stent was placed inside the artificial blood vessel. (A) An artificial vascular leg was identified in the abdominal aorta sac, and a covered stent was implanted. (B) Volume rendering image. The abdominal aorta had been replaced by an artificial covered stent.

**Figure 3:**
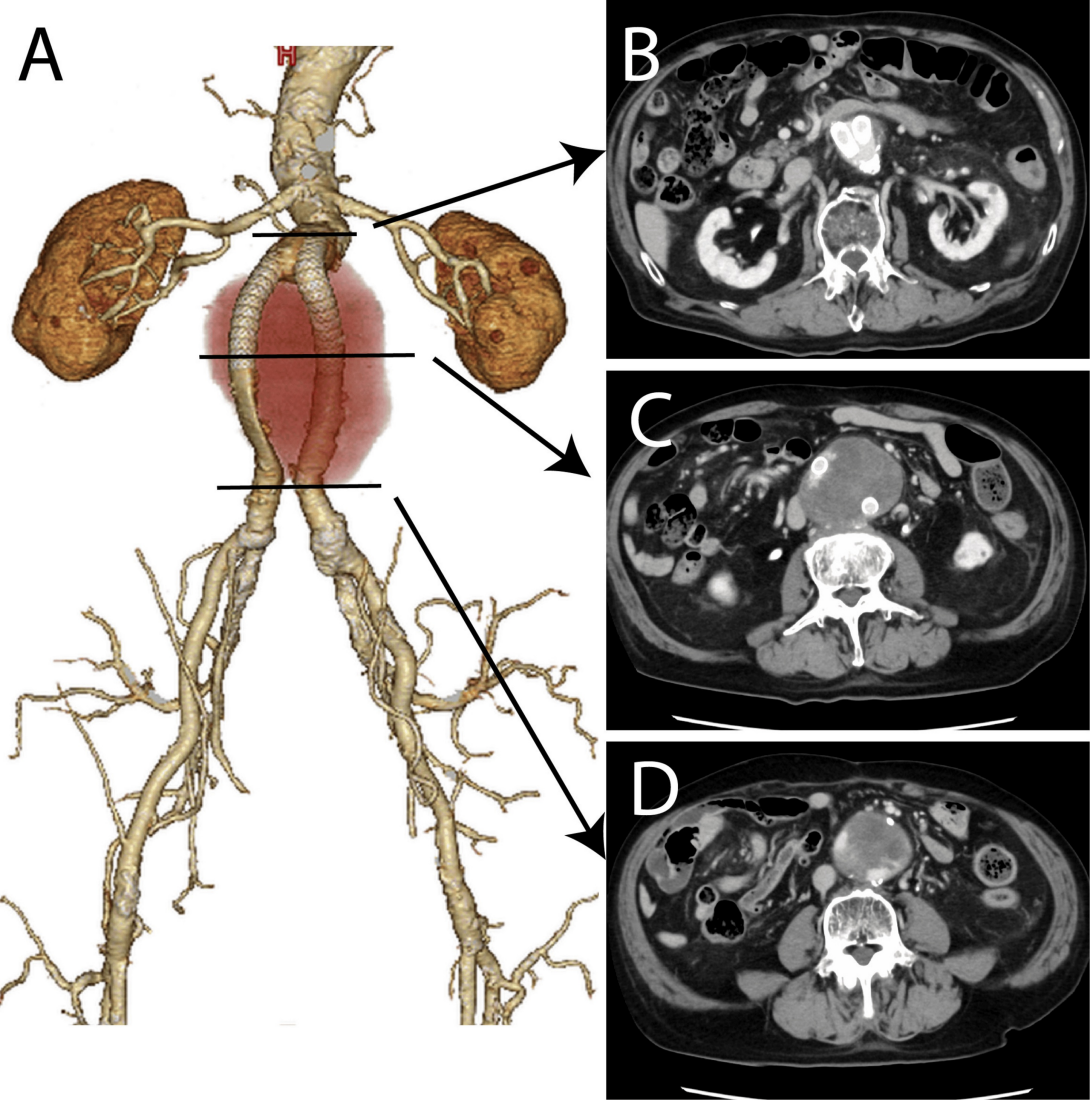
Contrast computed tomography image taken 37 years after artificial blood vessel replacement surgery in which a covered stent was placed inside the artificial blood vessel. (A) Volume rendering image. (B) A leak from an artificial blood vessel is visible at the proximal site. (C) A leak is visible at the end of the leg of the covered stent. (D) A leak from the artificial blood vessel is visible at the end of the artificial blood vessel.

During follow-up at a local hospital, the enlargement of the abdominal aortic sac was observed, likely caused by an endoleak from the synthetic graft. At 80 years of age, covered stents were placed bilaterally in the graft limbs (Figure 2). Despite this intervention, the abdominal aortic sac gradually enlarged over time (Figure 3). The patient was referred to our institution for further evaluation and treatment.

**Figure 4:**
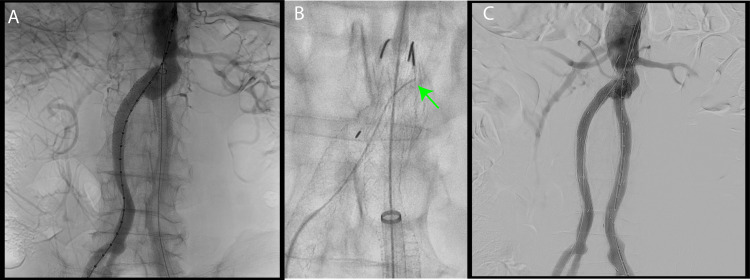
Intraoperative images taken during endovascular treatment. (A) Pre-angiography image revealing no leakage. (B) Aortic cuff placed at the proximal site (green arrow). (C) Post-angiography image revealing no leakage. The central anastomosis and distal anastomosis are covered by a stent graft.

The surgery was initiated under general anesthesia. An 18 Fr sheath was placed in the left femoral artery, while an 8 Fr sheath was placed in the right femoral artery. Contrast imaging revealed no apparent leak from the synthetic graft (Figure 4). Subsequently, a GORE® EXCLUDER® AAA Endoprosthesis aortic cuff (28.5/28.5/33; W. L. Gore & Associates, Inc., Flagstaff, AZ) was deployed just below the renal arteries. EXCLUDER® legs (2316140) were then placed bilaterally using the upside-down technique. Ballooning with the kissing technique was performed to achieve a D-shape configuration.

**Figure 5:**
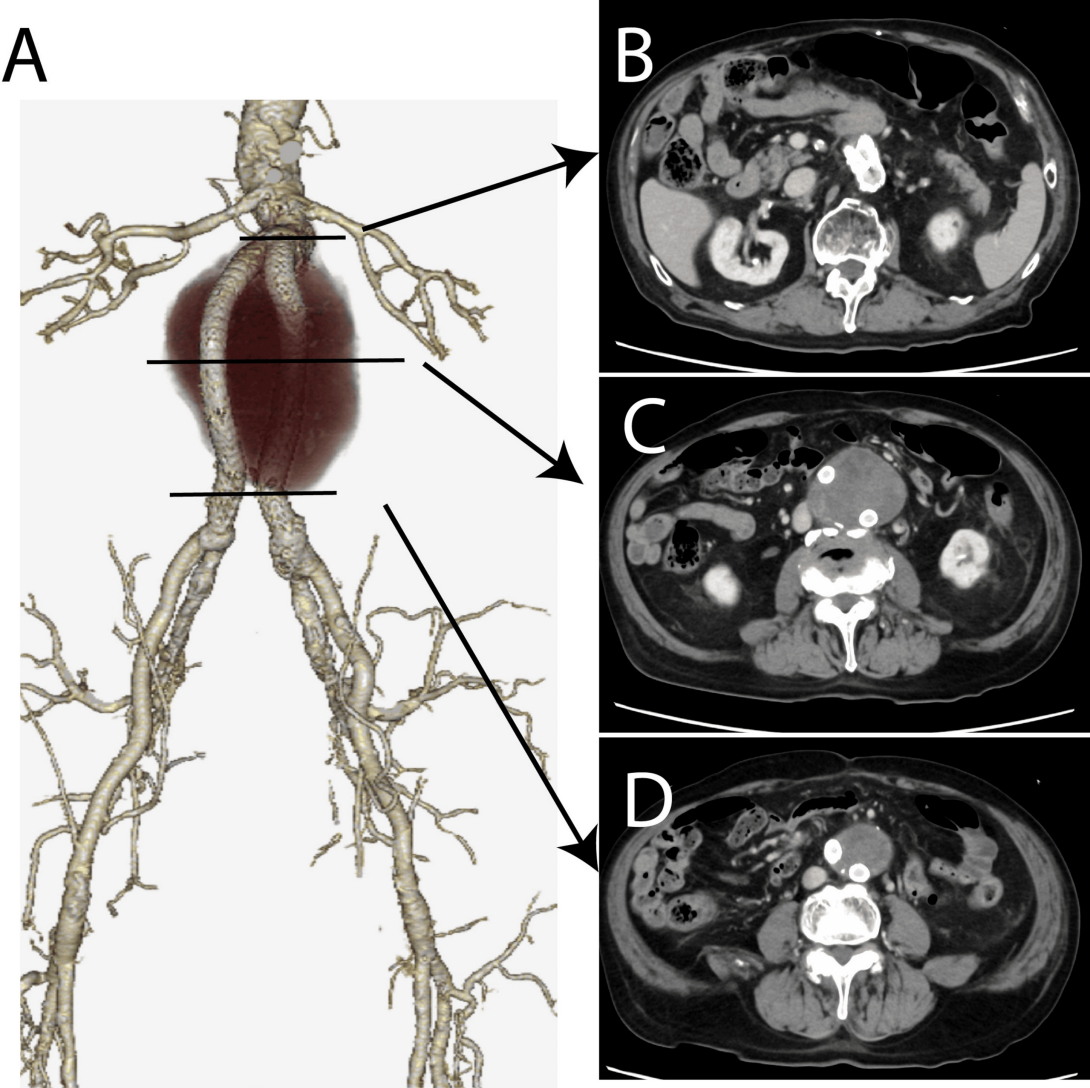
Contrast computed tomography image taken after endovascular therapy. (A) Volume rendering image. (B) No leak from an artificial blood vessel is visible at the proximal site. (C) Middle portion. (D) Distal site.

Additional EXCLUDER® legs (1616100 on the right and 1618100 on the left) were placed to cover the entire synthetic graft using the native vessel as the sealing zone. Final angiography confirmed the absence of leaks. On postoperative day 4, contrast-enhanced computed tomography (Figure 5) showed no residual leak. The patient was discharged on postoperative day 5 and remains under outpatient follow-up.

## Discussion

Synthetic grafts, commonly used in abdominal aortic replacement surgery, are widely considered durable and reliable for long-term use. However, evidence of their performance decades after implantation is sparse, with most studies focusing on outcomes within the first 10-15 years [5,6]. Synthetic graft failures, including structural degradation or endoleaks, have been reported at a low frequency, but these reports are often limited by their short follow-up duration. This case is particularly significant, as it documents synthetic graft failure at 37 years post-implantation, a rare occurrence that underscores the need for the extended monitoring of patients undergoing such procedures [7].

The management of synthetic graft failure typically involves open surgical replacement [8]. While this approach remains a definitive solution, it carries considerable risks, particularly in elderly patients or those with significant comorbidities. In this case, endovascular treatment was the first-line approach due to its minimally invasive nature. The decision to use the EXCLUDER® stent graft, which lacks a proximal bare stent, ensured that open surgical repair could still be performed if the endovascular treatment proved insufficient. This staged strategy reflects the flexibility and advancements in modern vascular surgical techniques, allowing for the safer management of complex cases such as this.

Despite the successful stent graft placement, the exact mechanism of graft failure could not be directly confirmed since no open surgical inspection was performed. Potential causes include gradual material fatigue, biomechanical stresses, or subclinical infection, all of which warrant further study. Understanding the precise factors leading to such late failures could provide valuable insights into improving synthetic graft design and long-term outcomes.

This case also highlights the importance of regular postoperative surveillance following abdominal aortic graft replacement. Scheduled imaging and clinical evaluations are crucial for the early detection of complications such as endoleaks, graft degradation, or aneurysm sac expansion. Long-term follow-up is particularly vital as more patients outlive the expected lifespan of their synthetic grafts, emphasizing the need for tailored guidelines to effectively address these late complications.

## Conclusions

Here, we encountered a case of abdominal aortic sac enlargement caused by leakage from a synthetic graft at 37 years post-implantation. The complete coverage of the synthetic graft with a stent graft was both feasible and successful. After the procedure, the leak from the synthetic graft resolved. Graft failure after 37 years is an exceptionally rare occurrence, prompting this report.
